# Is It Health or the Burial Environment: Differentiating between Hypomineralised and Post-Mortem Stained Enamel in an Archaeological Context

**DOI:** 10.1371/journal.pone.0064573

**Published:** 2013-05-29

**Authors:** Samantha McKay, Rami Farah, Jonathan M. Broadbent, Nancy Tayles, Sian E. Halcrow

**Affiliations:** 1 Faculty of Dentistry, University of Otago, Dunedin, New Zealand; 2 Department of Oral Sciences, Faculty of Dentistry, University of Otago, Dunedin, New Zealand; 3 Department of Oral Rehabilitation, Faculty of Dentistry, University of Otago, Dunedin, New Zealand; 4 Department of Anatomy, Otago School of Medical Sciences, University of Otago, Dunedin, New Zealand; University of Oxford, United Kingdom

## Abstract

Developmental enamel defects are often used as indicators of general health in past archaeological populations. However, it can be difficult to macroscopically distinguish subtle hypomineralised opacities from post-mortem staining, unrelated to developmental defects. To overcome this difficulty, we have used non-destructive x-ray microtomography to estimate the mineral density of enamel. Using a sample of deciduous teeth from a prehistoric burial site in Northeast Thailand, we demonstrate that it is possible to determine whether observed enamel discolourations were more likely to be true hypomineralised lesions or artefacts occurring as the result of taphonomic effects. The analyses of our sample showed no evidence of hypomineralised areas in teeth with macroscopic discolouration, which had previously been thought, on the basis of macroscopic observation, to be hypomineralisations indicative of growth disruption. Our results demonstrate that x-ray microtomography can be a powerful, non-destructive method for the investigation of the presence and severity of hypomineralisation, and that diagnosis of enamel hypomineralisation based on macroscopic observation of buried teeth should be made with caution. This method makes it possible to identify true dental defects that are indicative of growth disruptions.

## Introduction

Dental enamel is the most mineralised and hard tissue in the human body, and teeth are generally the most durable and well-preserved structure in the context of bioarchaeology (the study of people in the past from their skeletal remains). Therefore, they are considered to be a valuable means for assessment of the health of past populations [1]. As enamel formation can be disrupted by biological insults, and because it does not remodel over time, it provides a permanent record of past stresses that occurred during dental development [2]. The disruption of deciduous enamel production can be indicative of a stressful period in the mother’s life (during pregnancy) such as maternal metabolic disorders including diabetes, heart disease and kidney disease, but also more likely in past populations maternal viral and bacterial infections [3]. Other causes of hypomineralisation include exposure to tetracycline antibiotics and high levels of fluoride [4]. The formation of enamel occurs in two main phases, the secretion of the organic and inorganic matrix, and then the maturation phase where there is a break down of the organic component and the growth of the crystallites, with mature enamel being almost entirely mineral [4]. If disruption occurs during the mineralisation stage of enamel formation, this can result in hypomineralised enamel defects [3], which are evident as enamel opacities and discolorations [4].

Hypomineralised enamel can become discoloured/stained prior to emergence of a tooth into the oral cavity, as in the case of molar-incisor hypomineralisation, or subsequent to emergence, as in the case of fluorosis where stains can be ‘absorbed’ into opaque, porous enamel [4]. Also, pre-eruptively stained teeth can be stained futher post-eruptively. Post-mortem internal staining of enamel is visually similar to hypomineralised lesions and has long been known to occasionally result from the effects of the burial environment [5]. Acidic burial environments, for example, can lead to brownish discolouration in the dental enamel. Enamel may also be altered by the collapse and loss of dentine over time [5].

A high prevalence of what are believed to be developmental hypomineralisation defects or enamel opacities have been reported in the deciduous dentition of infants and children from prehistoric Southeast Asia [6]. However, there are problems in determining whether these are indicative of growth disruption, or are artefacts of the burial environment. Given that the bioarchaeological study of infants and children is increasingly becoming recognised as a sensitive indicator of biological and social life in the past [7]–[10], a means of establishing the cause of these defects in archaeological samples is needed.

In contrast to true hypomineralisation defects, post-mortem staining is not expected to show any decrease in mineral density. Therefore, determining the mineral density of enamel can contribute to distinguishing post-mortem staining from hypomineralisation defects. For living patients Farah et al. [11] recently showed x-ray microtomography (XMT) to be a valuable tool in detecting the decrease in mineral density of visually pronounced, true hypomineralised enamel defects.

The aim of this research was to use XMT to examine the mineral density of dental enamel of deciduous teeth collected from Ban Non Wat (a late prehistoric site in the Upper Mun River Valley in northeast Thailand) with and without enamel staining, and use these findings to investigate whether these stains are more likely to have occurred post-mortem or be true developmental enamel hypomineralisation. We show that it is possible to exclude post-mortem effects on enamel when determining the prevalence of enamel defects to investigate possible growth disruptions and childhood stress in past populations. Importantly, for archaeological samples, the method is non-destructive.

## Materials and Methods

### Materials

**Figure 1:**
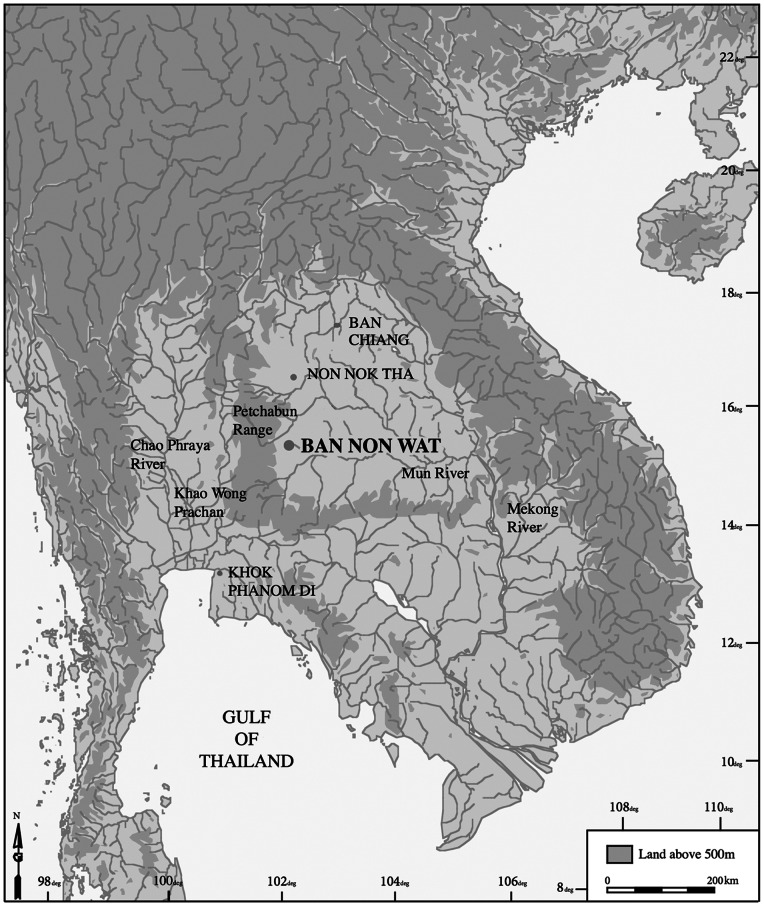
The site of Ban Non Wat in northeast Thailand.

Nineteen deciduous teeth were collected from infant and child burials from the prehistoric site of Ban Non Wat (3800-1500BP), situated in the Upper Mun River Valley in northeast Thailand (Fig. 1). This site was excavated under the Origins of Angkor Archaeological Project, directed by Professor Charles Higham (University of Otago) and Dr Rachanie Thosarat (formerly Thai Fine Arts Department) [12]. The skeletal samples are housed at the Thai Fine Arts Department bone storage facility in Phimai, Northeast Thailand. All necessary permits were obtained for the described study, which complied with all relevant regulations. Permissions were obtained from the National Thai Research Council and the Thai Fine Arts Department to study these human remains. One tooth was assessed from each of 19 burials. Ten teeth displayed no macroscopic evidence of enamel discolouration and formed the control or ‘unaffected’ group in this study (Table 1). Nine teeth had macroscopic areas of enamel discolouration and served as the affected group. No treatment or preparation of the enamel was carried out before examination of the teeth.

**Table 1:** Characteristics of discoloured and non-discoloured teeth.

Burial Number	Tooth*	Affected by discolouration	Type of defect	% of tooth surface affected
19	63	unaffected		
44	53	affected	Demarcated	<1/3
84	73	affected	Diffuse	<1/3
116	83	unaffected		
130	63	affected	Demarcated	<1/3
141	63	unaffected		
146	63	affected	Diffuse	<1/3
165	83	affected	Diffuse	<1/3
213	73	unaffected		
274	63	affected	Demarcated	<1/3
297	73	unaffected		
381	63	affected	Demarcated	<1/3
426	53	unaffected		
519	63	unaffected		
541	52	unaffected		
548	53	unaffected		
559	72	affected	Demarcated	<1/3
563	53	unaffected		
584	83	affected	Demarcated	<1/3

* The nomenclature used for recording teeth was the Fédération Dentaire Internationale (FDI) two-digit system [13]. In this scheme the first digit specifies the quadrant of the mouth and the second digit is the tooth in each quadrant. Each set of dentition is separated into four quadrants. For the deciduous dentition, the four quadrants and hence the first digits of the recording scheme are five to eight, ‘5’ being the right maxillary, ‘6’ the left maxillary, ‘7’ the right mandibular and ‘8’ the right mandibular quadrant. Within every quadrant, individual deciduous teeth are numbered one to five progressing from the mesial to the distal part of the mouth. For example, the deciduous left mandibular central incisor is 71.

### Macroscopic Appearance of the Teeth

The Developmental Defects of Enamel (DDE) Index is a method of objectively categorising a wide range of enamel defects at the tooth level on the basis of the appearance, size, distribution, and location of defects [14]–[19]. Defects were categorised as ‘demarcated’ (code 1) or ‘diffuse’ on the basis of macroscopic appearance (code 2). The extent (or severity) of each defect was categorised as less than 1/3 of the tooth surface (code 1), at least 1/3 but less than 2/3 (code 2), and at least 2/3 (code 3) [20]. All teeth were photographed and all defects classified by SH.

### X-ray Microtomography Scanning

**Figure 2:**
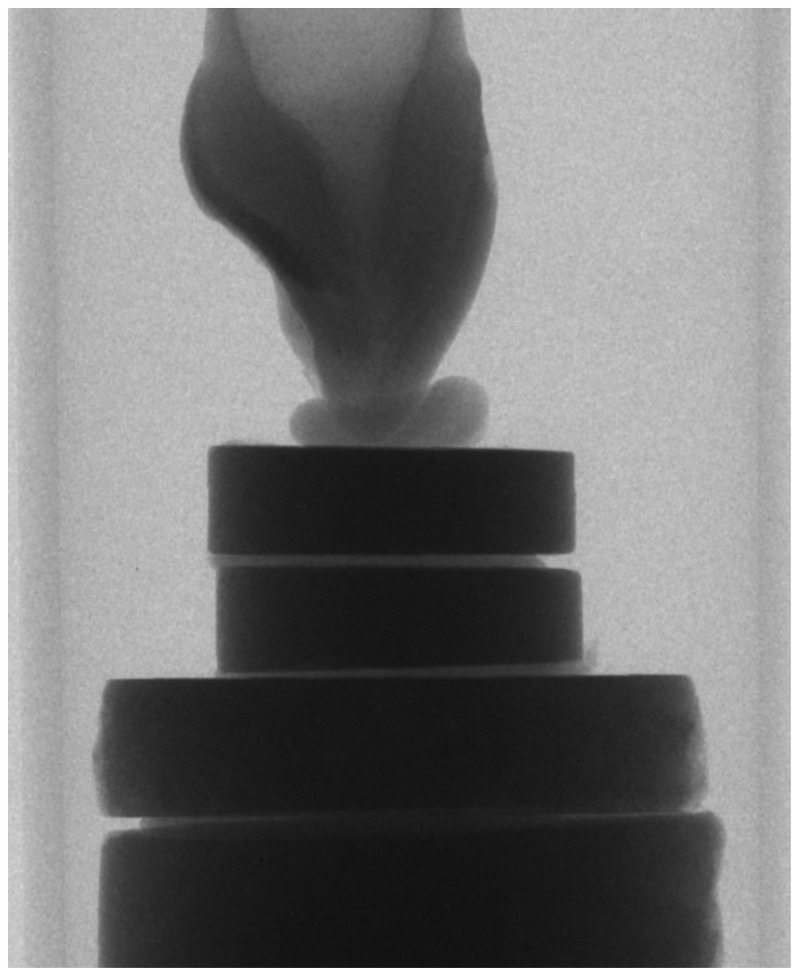
Typical projection of a study tooth on top of the four phantoms.

X-ray microtomography is an imaging method used to reconstruct a small object through multiple x-ray projections. XMT scanning (SkyScan 1172, SkyScan N.V., Aartselaar, Belgium) was used to quantify the mineral density of each tooth in the sample. Cross-sections of each tooth were reconstructed using a modified Feldkamp cone-beam algorithm (NRecon, Version 1.5.1.4). The mineral density of enamel was calculated by comparison with scans of discs of homogenous hydroxyapatite structure (Fig. 2), or ‘phantoms’ of known mineral density following Farah et al. [11]. The densities of the phantoms (1.85 g/cm^3^, 1.88 g/cm^3^, 3.04 g/cm^3^, and 3.16 g/cm^3^) were calculated at the beginning of this study by dividing weight by volume. The mineral density of each disc of four hydroxyapatite phantoms is plotted against its grey level recorded from the XMT scan. The plot is then used as a standard curve to calculate the mineral density of enamel depending on its greyscale level recorded by the XMT scan.

### Scanning to Detect Mineral Density

**Figure 3:**
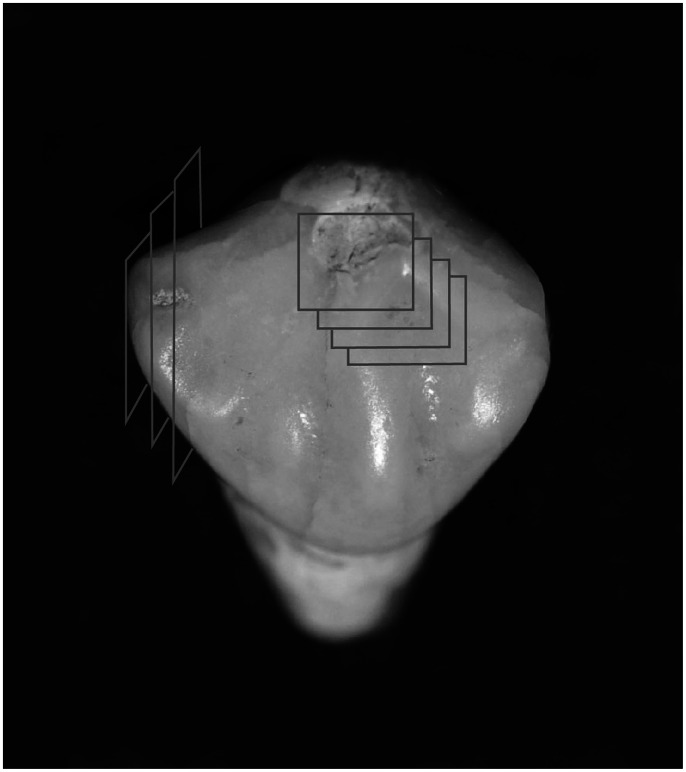
Schematic representation of the locations of the XMT cross-sections through a tooth.

In order to map the mineral density distribution in the sample teeth, multiple readings were taken of each of the phantoms and the nineteen teeth in the study (Fig. 3):

- In each of the four hydroxyapatite phantoms, three horizontal cross-sectional slices were chosen and a grey level reading for the circular disc shape was recorded. The outer few pixels (5–10 micrometers) were not included when recording the grey level reading in order to reduce error due to partial volume effect, an issue arising when a single pixel/voxel represents more than one tissue or the tissue and the dark background resulting in reduced mineral density being recorded [21]. The three readings per disc were averaged and recorded.
- In each tooth, horizontal cross-sectional slices of enamel were taken at 1 mm intervals from the cementum-enamel junction (CEJ) to the cusp/incisal tip of the tooth. Within each slice, four grey level measurements were recorded by choosing a ‘region of interest’ (ROI) at four different locations in the enamel; the mesial and distal aspect of the buccal, and the mesial and distal aspect of the lingual. Each ROI location was kept as consistent as possible in each slice. The mean of the four grey level readings within each slice was calculated and recorded as the mineral density of enamel at each level. Care was taken in the selection of each ROI to ensure the outer and inner few pixels of enamel were not included, in order to minimize the risk of bias due to the partial volume effect.
- In each tooth sample, one horizontal cross-sectional slice was chosen in order to record the grey level readings from the dentine-enamel junction (DEJ) to the outer enamel surface. In control teeth this cross-sectional slice was located approximately mid-way between the CEJ and the cusp/incisal tip, where the enamel was of an adequate thickness. For the affected teeth, electronic callipers were used to visually measure the distance from the centre of the enamel defect to the CEJ so a horizontal cross sectional slice of the scan could be taken at the level where the enamel defect appeared macroscopically. In each cross sectional slice, depending on the thickness of each tooth’s enamel, four to five 5×5 pixel ROIs were chosen, 100 µm apart. The aim of this step was to determine grey scale readings, and hence the mineral density, for the entire enamel thickness, therefore a partial volume effect may be associated with the first and last readings.

### Minimising Error during Scanning

During scanning, ring artefacts can be caused by inhomogeneities in the x-ray detector. These can be minimised by randomly moving the object throughout the scanning process [22], and this was done by setting the Ring Artefact Correction to a setting of 20 during reconstruction of the image.

The use of polychromatic x-rays, as in the system used in this experiment, may allow beam hardening to be exhibited [23], where x-rays of lower intensity are attenuated at the outer part of the scanned object, thus creating a false impression of lower density centrally and a higher density at the surface. To overcome this problem, metal filters are installed in the path of the beam to cut away the low-energy x-rays, and mathematical corrections during the reconstruction of the image are used effectively. Here the beam hardening correction (%) was set between 70–80.

### Mineral Density Equations

**Figure 4:**
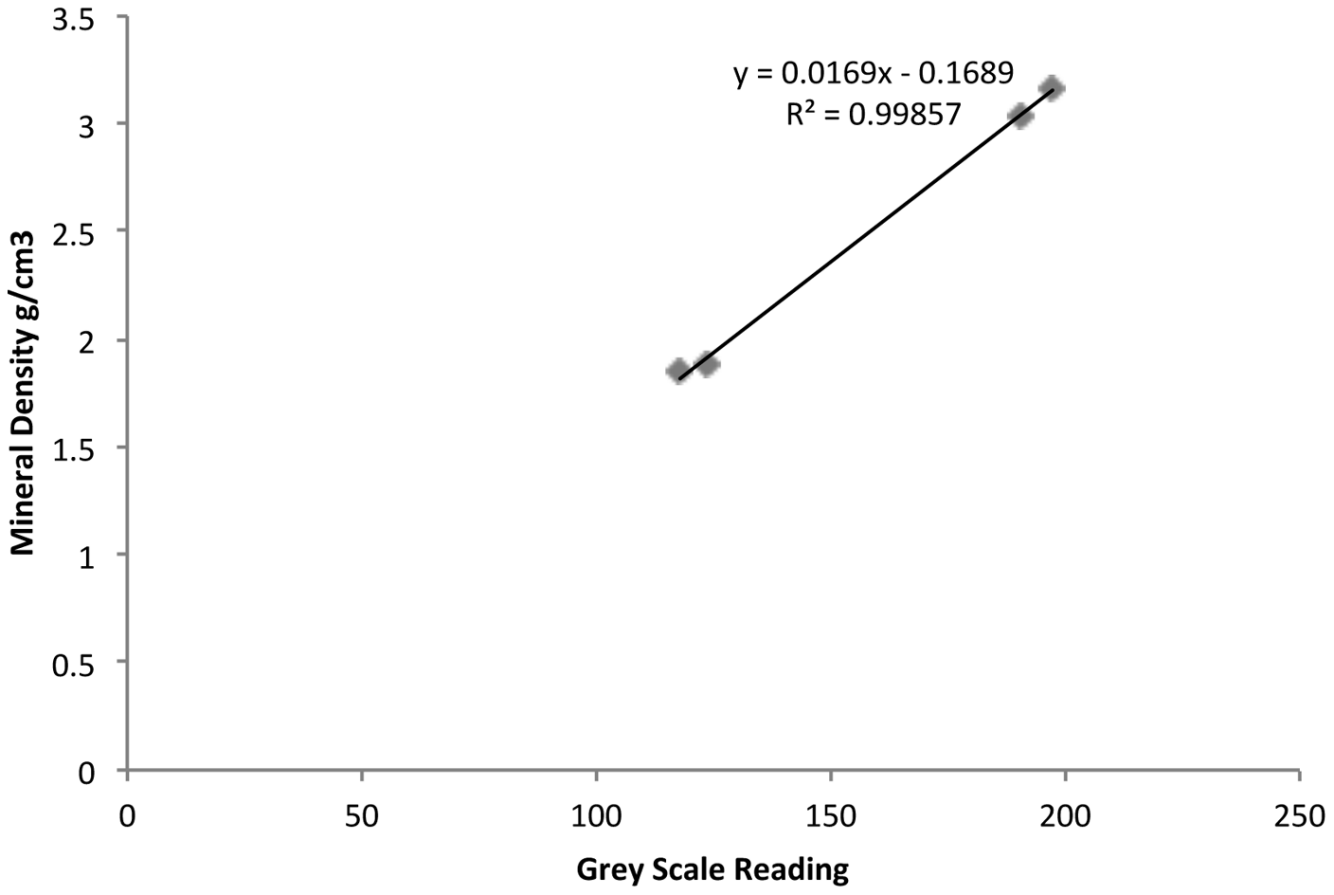
Typical scatter-plot of the four phantoms (burial 274).

To calculate the mineral density of the phantoms and the regions of interest in the enamel, the software package CT Analyser (Version 1.5.0.0, SkyScan N.V., Aartselaar, Belgium) was used. By plotting each hydroxyapatite phantom disc’s grey scale reading against the previously calculated mineral density, a scatter plot with linear regression equation was generated. Figure 4 is a typical projection showing the four phantoms with one of the teeth in the study. The equation of this graph was then used to calculate the mineral density of each region of interest within the enamel.

### Statistical Analyses

Data were analysed in Intercooled Stata Version 10 (StataCorp, Texas). Differences in mineral density were tested for statistical significance using unpaired t-tests.

## Results

**Figure 5:**
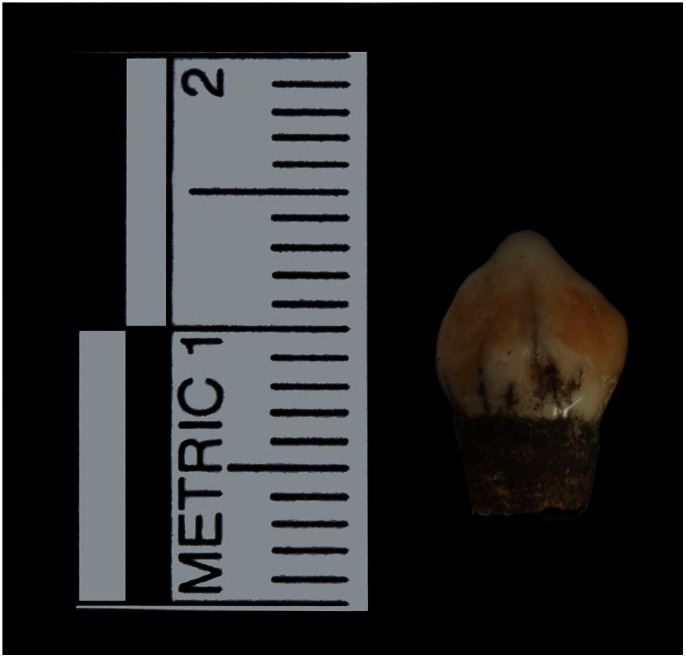
Example of an affected tooth (burial 146; tooth 63; DDE type code: 2; extent code: 1).

All the enamel defects in this study were either demarcated (code 1) or diffuse opacities (code 2), and covered less than 1/3 of the total enamel surface area of the tooth (code 1) (e.g., Fig. 5) (Table 1).

### Measurement of Mineral Density from the CEJ to the Cusp/Occlusal Tip

**Figure 6:**
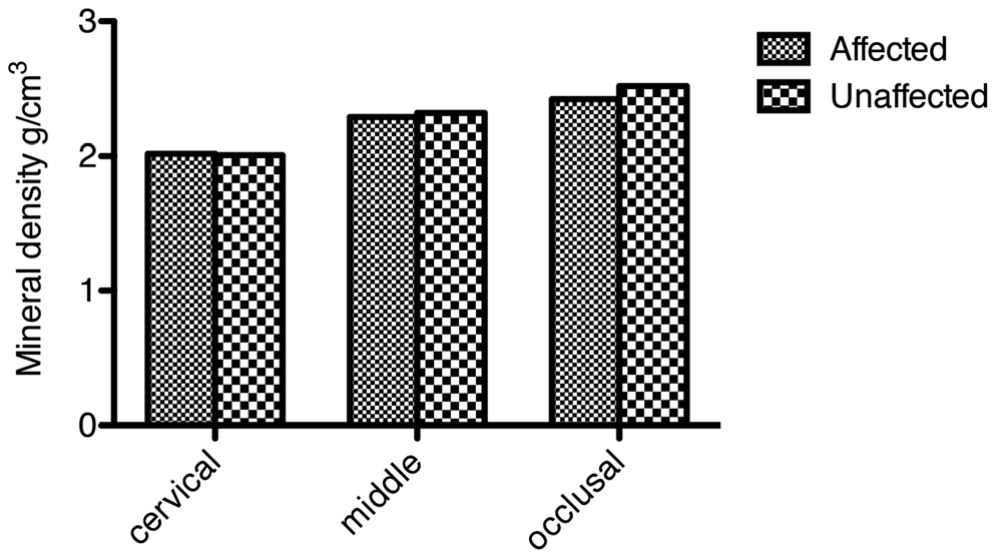
Mineral density from the cervical third to the occlusal third in the affected and unaffected teeth.

The average mineral density readings for each horizontal cross-sectional slice were divided into three categories; ‘cervical third readings’, ‘middle third readings’, and ‘occlusal third readings’. The average mineral density of the cervical third in the affected teeth was 2.02 g/cm^3^ (SD = 0.13), the middle third it was 2.30 g/cm^3^ (SD = 0.10), and the occlusal third it was 2.42 g/cm^3^ (SD = 0.07) (Fig. 6). The control teeth showed the same general gradient with the mineral density being highest in the occlusal third and lowest in the cervical third. The average mineral density readings for the control teeth were 2.01 g/cm^3^ (SD = 0.13) cervically, 2.32 g/cm^3^ (SD = 0.09) in the middle third and 2.53 g/cm^3^ (SD = 0.08) in the occlusal third. There were no statistically significant differences in the average mineral densities of the affected enamel and the control enamel in the cervical and middle third, but the mineral density averages in the occlusal third did differ significantly, with the mineral density of the affected teeth being less mineralised (p<0.05).

### Measurement of Mineral Density from the Dentine-enamel Junction to the Outer Surface of Enamel

**Figure 7:**
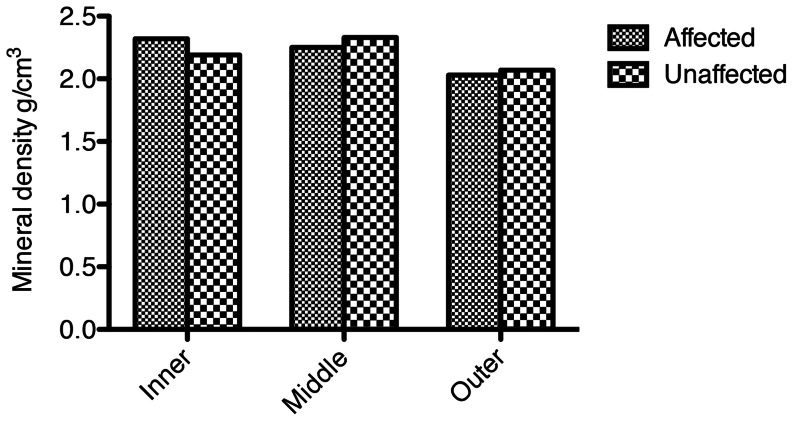
Mineral density from the DEJ third to the outer third in the affected and unaffected teeth.

The mineral density readings spanning from the DEJ to the outer surface of enamel were also grouped into three categories, the ‘inner third’, ‘middle third’, and ‘outer third’. In the affected teeth the average mineral density of enamel in the inner third was 2.32 g/cm^3^ (SD = 0.20) (Fig. 7). This decreased to 2.26 g/cm^3^ (SD = 0.30) in the middle third, and to 2.03 g/cm^3^ (SD = 0.29) in the outer third of enamel. The average mineral densities of the control teeth showed a slightly different gradient with an initial inner third reading of 2.20 g/cm^3^ (SD = 0.21), which then increased to 2.34 g/cm^3^ (SD = 0.20) in the middle third, before decreasing to 2.07 g/cm^3^ (SD = 0.22) in the outer third. There were no statistically significant differences among the mineral densities of the affected and control teeth in all three categories (Table S1).

## Discussion

This research has shown that it is possible to differentiate hypomineralised lesions from post- mortem staining in teeth by determining the mineral density of the enamel. It has also shown that it is important to use an objective measure such as XMT when determining if true hypomineralised lesions exist, especially if the lesions are classified on the lower spectrum or non-hypoplastic lesions within the DDE Index [20], which do not affect the quantity of enamel.

There was no evidence of hypomineralisation in these affected teeth. Farah et al. [11] reported that hypomineralised defects show approximately 19% lower mineral density in comparison with sound enamel. In our samples, the mineral density readings of the affected teeth sampled were within the limits of normal mineral density [11]. Although we found a statistically significantly lower mineral density in the cuspal third of the crown in the affected compared with the control teeth, given that it was only the cuspal third that had statistically significant lower mineral density, and that the areas of discolouration were not limited to this area, we interpret this difference in mineral density as being unrelated to the discoloration. Both these findings indicate that the discolorations on all of the affected teeth cannot be classified as true hypomineralised lesions. The affected teeth were of slightly lower mineral density, indicating that their structure may be more porous, and perhaps more prone to absorb stains from their surroundings compared with the control teeth.

A positive gradient in mineral density of enamel was observed from the CEJ to the cusp/incisal tip in both the affected and control teeth. This is in agreement with previous studies [24], [25]. The more mineralised cusp tips may have developed in order to meet their functional role of mastication in the oral cavity [11]. It should be noted that the lower mineral density reading at the cervical region may have been influenced by the partial volume effect.

From the DEJ toward the outer surface, the mineral density in the affected teeth decreased, whereas from the DEJ towards the outer surface, the mineral density in the control teeth initially increased and then decreased, with the lowest density being found at the outer aspect. Previous studies have reported a gradient of increasing mineral density in sound teeth [25], [26]. Others have shown the enamel closest to the DEJ having the lowest mineral density reading, but the middle third being somewhat more mineralised than the outer third [27]. The affected teeth in this study showed a slightly different gradient again, with the most mineralised enamel being closest to the DEJ. The control teeth followed the pattern described by Clementino-Leudemann and Kunzelmann [27], where the middle enamel has the highest mineral density. This can possibly be explained through evidence from a study carried out by Robinson et al. [26], which showed the middle part of enamel to have higher calcium and phosphate ion concentration. The partial volume effect may have contributed to the low mineral densities recorded in the outermost thirds. Another possible explanation is that a low mineral density at the outer surface may indicate that the tooth was unerupted or newly erupted, and the outer surface was simply still undergoing post-eruption maturation. A third possibility is that the decrease in mineral density at the outer aspect of the enamel may be a result of ion exchange between the enamel and the burial environment. Although there has been little research into the effects of burial and possible ion-exchange on enamel mineral density, ion-exchange may be a feasible explanation.

Diagenesis is any change undergone by a tissue after its burial, and the two most important environmental factors which affect this are soil composition and the nature of ground water. The presence of water in the environment is likely to increase the affinity for ions such as fluoride, the presence of metal or metal grave goods may introduce metal ions into the tooth specimen, and shell middens may provide carbonate ions which may be incorporated into the mineral lattice [28].

The burial site at Ban Non Wat was affected seasonally by monsoonal rainfall, and the presence of water may have affected the mineral density of surface enamel. The lower mineral density adjacent to the DEJ in sound teeth may be related to higher amounts of magnesium, carbonate and proteins that are located near the DEJ due to the narrower and decussated nature of the enamel rods [26]. Although not able to be proven, perhaps the lower mineral density at the outer surface in the affected teeth compared with the control teeth could be the result of a more porous structure, one that is more likely to absorb material, resulting in post-mortem staining.

### Conclusions

Despite each of these discolorations having initially been classified as enamel hypomineralisation (on the basis of macroscopic appearance), we found the mineral densities in the enamel of the teeth affected by discolouration were within the normal limits for sound enamel and were comparable to the mineral densities of the teeth not affected by discolouration. Our findings provide no evidence of hypomineralisation in this sample, and all enamel discolorations were most likely due to post-mortem changes. Our findings suggest that macroscopic observation is not always a reliable method of differentiating between post-mortem stains and enamel hypomineralisation in bioarchaeological samples. We recommend that a non-destructive method for estimation of mineral density, such as XMT, be used to investigate the mineral density of enamel below any discolouration.

## Supporting Information

Table S1 **Comparison of mineral density (g/cm^3^) of discoloured and non-discoloured teeth.**
(DOCX)
