# Supplementary material for: Is It Health or the Burial Environment: Differentiating between Hypomineralised and Post-Mortem Stained Enamel in an Archaeological Context
Source: PLoS One. 2013 May 29;8(5):e64573. doi: 10.1371/journal.pone.0064573 (PMC3667180; doi:10.1371/journal.pone.0064573)
Supplement: Table S1 — Comparison of mineral density (g/cm3) of discoloured and non-discoloured teeth. (DOCX) [file pone.0064573.s001.docx]

Supplementary Table 1. Comparison of mineral density (g/cm^3^) of discoloured and non-discoloured teeth

|  | **Unaffected teeth**  **Mean (95% CI)** | **Affected teeth**  **Mean (95% CI)** |
| --- | --- | --- |
| Region of interest, cervical third | 2.01 (1.96, 2.06) | 2.02 (1.95, 2.09) |
| Region of interest, mid third | 2.32 (2.29, 2.36) | 2.30 (2.24, 2.36) |
| Region of interest, cuspal third | *2.52 (2.46, 2.57)* | *2.42 (2.37, 2.47)* |
| Horizontal slice, DEJ third | 2.20 (2.05, 2.35) | 2.29 (2.14, 2.44) |
| Horizontal slice, middle third | 2.33 (2.24, 2.43) | 2.26 (2.03, 2.48) |
| Horizontal slice, surface third | 2.04 (1.88, 2.20) | 1.96 (1.73, 2.19) |

Statistically significant differences shown by italics
